# The Omentum—A Forgotten Structure in Veterinary Surgery in Small Animals’ Surgery

**DOI:** 10.3390/ani14131848

**Published:** 2024-06-21

**Authors:** Magdalena Morawska-Kozłowska, Aleksandra Wilkosz, Yauheni Zhalniarovich

**Affiliations:** Department of Surgery and Radiology with Clinic, Faculty of Veterinary Medicine, University of Warmia and Mazury in Olsztyn, 10-719 Olsztyn, Poland; aleksandra.wilkosz@uwm.edu.pl (A.W.); eugeniusz.zolnierowicz@uwm.edu.pl (Y.Z.)

**Keywords:** omentum, soft tissue surgery, omentalization in small animals

## Abstract

**Simple Summary:**

The omentum is a fold of peritoneum, a membrane that lines the abdominal cavity and covers the abdominal organs. It consists of two main parts: the greater omentum and the lesser omentum. The greater and lesser omentum play important roles in the function and structure of the abdominal cavity, including providing support and protection for organs, storing fat, and contributing to immune responses. Over the years, it has been forgotten by modern science and clinicians. The following article presents the current knowledge about using this structure in veterinary surgery.

**Abstract:**

The greater and lesser omentum are derived from embryonic mesogastrium. The expansive greater omentum in dogs covers intestinal coils, while in cats, it is smaller. Comprising distinct portions, the greater omentum is rich in lymphatics and blood vessels. Conversely, the lesser omentum spans the liver, stomach, and duodenum. Studies on canine omentum reveal unique immune cell composition and regenerative potential attributed to adipose tissue-derived stromal cells (ADSCs). These cells hold promise in regenerative medicine, showing enhanced abilities compared with ADSCs from other sources. The omentum is critical in tissue repair and pathology, making it invaluable in veterinary surgery across various medical fields. The aim of this article was to research current knowledge about the applications of the omentum in veterinary surgery and the possibilities of using this structure in the future.

## 1. Introduction

The embryonic dorsal and ventral mesogastrium are the source of peritoneal sheets called the greater and lesser omentum (Omentum majus and Omentum minus) [1,2,3]. They are loaded with fatty streaks of lymphatics and blood vessels. The greater and lesser omentum encircle the canine omental bursa, essentially a virtual cavity [4,5]. The greater omentum in dogs is an incredibly huge structure that covers the intestinal coils bilaterally and ventrally as it stretches from the stomach to the urinary bladder in a double-folded fashion. At the same time, in cats, it is a shorter structure. There are three parts to the greater omentum: the bursal, splenic, and veil portions [1,2,6,7]. The lesser omentum is not very large and not highly complex. It crosses the region bordered by the liver, the stomach’s smaller curvature, and the duodenum’s cranial portion [1]. It is separated into the hepatogastric ligament (Ligamentum hepatogastricum) and the hepatoduodenal ligament (Ligamentum hepatoduodenale) based on the attachment point. The latter contains the hepatic artery, the portal vein, and the common bile duct [4,8].

Despite its frequent use in surgery, the omentum remains largely unexplored and underrated as an organ [9]. Surprisingly, few studies have delved into its structural organization in companion animals. Many publications rely on data from humans, laboratory animals, or structures like the pleura or peritoneum [10]. In 2016, a thorough analysis of the tissue composition in the omentum of clinically healthy dogs was carried out [11]. This structure was identified at the macroscopic level in two different zones: the translucent and adipose-rich regions, just like in other mammals [12,13,14]. The omentum included a single layer of mesothelial cells atop a submesothelial layer primarily composed of collagen fibers and fibroblast-like cells, with a significant presence of fat cells in adipose-rich areas. However, regarding the composition and structure of the immune population, findings in normal canine omentum diverge from those in other species [10,14,15,16]. Notably, no visible OALT (Omental-Associated Lymphoid Tissue) was observed, and lymphoid cell aggregates were absent. However, minimal immune cell presence was noted in translucent regions, primarily in neutrophils and macrophages. In the adipose-rich areas, immune cell presence was exceedingly scarce, with both macrophages and neutrophils notably absent. Only a minimal presence of CD45+ cells was detected in a few samples. Electron microscopic studies revealed the omentum’s sieve-like structure, with fenestrations varying in size across samples. The function of these fenestrations remains debated, with suggestions ranging from facilitating adhesion formation to enabling fluid transport. Observations also showed significant pinocytotic activity in mesothelial cells, with the presence of microvilli whose exact function is yet to be determined [11]. 

The omentum has significant regenerative potential since it is an organ rich in mesenteric stem cells [17]. Adipose tissue-derived stromal cells (ADSCs) have been isolated from both dogs and cats in fat-rich regions of this organ [18,19,20,21,22,23,24,25,26]. These cells are located in the omentum of canines and cats and have sparked interest in their potential in regenerative medicine [27,28]. Studies indicate that ADSCs extracted from the omentum possess distinct qualities, such as a heightened ability to multiply and specialize, compared with ADSCs sourced from other adipose tissues. These cells have demonstrated encouraging outcomes in early-stage investigations for various therapeutic uses, including repairing and renewing tissues [29,30]. Exploiting the regenerative capacities of omental-derived cells shows great potential in advancing veterinary medicine in small animal practice, particularly in innovating treatments for canine conditions that necessitate tissue regeneration [31]. In dogs, ADSCs from omentum are expressed in CD90, CD44, CD29, CD105, CD13, CD133, CD73, and CD31 [19,20,22,23,24,25,32], and in cats, in CD90, CD44, CD 29, CD105, CD73, CD9, and MHC class I was found in the omentum structure [27,33,34,35,36,37,38,39,40]. 

The omentum’s role in the abdominal cavity is versatile. It participates in both repair and pathological processes, e.g., in the formation of ovarian cancer in women. According to the reference, the spread was driven by tumor-associated macrophages (TAMs) in the omentum, which served as the premetastatic niche [41]. Omentum is a specific tissue that reacts to inflammation [42], attaches firmly to the wound closure site [43], and participates in the healing process. Tissue damage sets off a series of inflammatory and matrix remodeling processes that are required to restore tissue integrity and function [44]. The omentum’s morphology and physiology make it an invaluable instrument for a range of intra- and extra-abdominal surgical procedures in various medical specialties, including neurosurgery, soft tissue surgery, oncology, and plastic surgery [45,46,47]. In veterinary medicine, in small animal surgery, there are reports of the use of omentum in treating pancreatic diseases, abscess healing, wound healing, bone healing, prostatic diseases, and the treatment of cysts [48,49,50,51,52,53,54,55,56].

The aim of this article was to research current knowledge about the applications of the omentum in companion animals’ surgery and the possibilities of using this structure in the future. 

## 2. Materials and Methods

**Figure 1:**
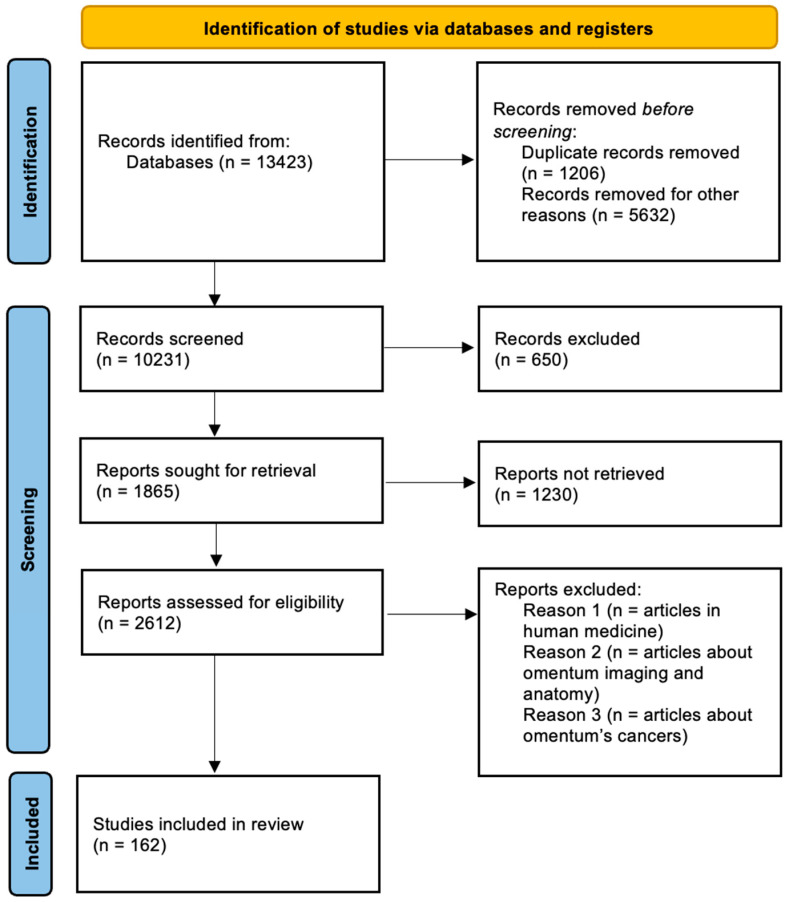
The PRISMA flow diagram illustrates the article selection process, documenting the inclusion and exclusion of articles at each stage. This ensures transparency and thoroughness in identifying the 162 articles that were ultimately included in the review.

A systematic literature review was conducted to collect information on using the omentum in small companion animal veterinary surgery, following the Preferred Reporting Items for Systematic Reviews and Meta-Analyses (PRISMA) guidelines (Figure 1). 

The search strategy included electronic databases such as PubMed, Web of Science, and ScienceDirect to identify relevant articles published from 1939 to 2023. Search terms were carefully selected to cover a wide range of pertinent topics, including variations of “omentalisation in dogs”, “omentalisation in cats”, “omentum”, “omentum in surgery”, “greater omentum in surgery”, “omentalisation”, and related terms.

A total of 162 articles were meticulously selected for inclusion in this review based on their relevance and contribution to understanding the role of the omentum in companion animal surgery. Systematic data extraction was conducted, focusing on key elements such as the use of the omentum in the treatment of abscesses and cysts in body cavities, its application in wound treatment, its regenerative and deranged abilities, and the cellular composition that provides these functions.

Selected articles underwent a critical evaluation for methodological rigor. Parameters assessed included the study design and the exclusive use of the omentum in surgeries involving dogs and cats.

## 3. Omentum in the Treatment of Pancreatic Diseases

In veterinary medicine, there are few reports of omentalization in pancreatic pathologies. Available literature documents treatment via the omentalization of pancreatic pseudocysts and abscesses in dogs [50,51]. There are also cases of omentalization in cats [57,58]. Pancreatic pseudocysts are more popular findings in dogs than in cats [57]. Usually, the occurrence of pseudocysts or abscesses in the pancreas is associated with pancreatitis [58,59,60,61,62,63,64,65]. Procedures performed on the pancreas and in its area can be challenging due to the danger of releasing autolytic pancreatic enzymes. Pancreatitis can influence intra- and postoperative complications. However, in patients without coexisting diseases, the prognosis is positive. The most common indications for the surgical approach for pancreatitis in animals are its severe symptoms, peritonitis, or extrahepatic biliary obstruction [58,66]. Using omentum as a dressing to promote better healing can help patients recover faster.

The earliest publication about surgical management by pancreas omentalization describes the treatment of pancreatic pseudocyst in a 3-year-old male Labrador Retriever. Based on the anamnesis and clinical examinations, the dog was diagnosed with acute pancreatitis. The pharmacological treatment was introduced. After the recurrence of symptoms, the dog returned for further investigation. A 3 cm cystic structure in the right lobe of the pancreas was diagnosed using ultrasound. To confirm the suspicion of pancreatic pseudocyst or abscess, an exploratory laparotomy was recommended. During the surgery, a cyst was found, and the wall of the cyst was incised to collect samples for histopathology. The omentum was then sutured in place over the cystic wall. The 3-0 polydioxanone simple interrupted sutures were used. After one month post-surgery and then after seven months, the patient was brought in for the postoperative evaluations. The ultrasound examinations showed no signs of pancreatic cyst during both visits [50].

Johnson and Mann, in their study, compared postoperative outcomes in dogs with pancreatic abscesses treated with omentalization and abdominal closure to dogs treated with open peritoneal drainage. In eight of the twelve dogs reported in that study, a surgeon performed omentalization with abdominal closure. Five of them survived and were able to recover. Two of the three dogs that did not survive had already preexisting health conditions, such as lymphosarcoma, megaoesophagus with aspiration pneumonia, and hyperadrenocorticism. In a group of four dogs with an open peritoneal drainage method, omentalization was used additionally in two of these dogs, and partial omentectomy was performed in one. Unfortunately, none of them survived the procedures. There were no reported complications in three of the dogs where omentalization was performed [51]. 

Pancreatic cysts in cats are less common findings. Bartner and Viviano described the case of a cat with multiple recurrent pancreatic cysts recognized using ultrasound and computed tomography. The decision was made to perform exploratory laparotomy for the resection of cystic structures. One of the findings was a multilobulated structure filled with fluid connected with the pancreas and proximal duodenum. The surgeon drained, opened, and, at the end, omentalized the structure. The patient’s recovery was successful. After 1 month following surgery, the cat returned with lethargy and loss of appetite. A reoccurrence of pancreatic cysts with progressive pancreatic atrophy was discovered. Blood results indicated non-ketotic hyperosmolar syndrome and hemoconcentration. The cat’s condition got worse over the days, and the owners decided to euthanize it. Pancreatic inflammation and atrophy could be associated with fast disease progression, which results in diabetes mellitus [57].

There is one existing study about the laparoscopic omentalization of pancreatic cysts in a cat [20]. It was performed during explorative laparoscopy, following a finding of cystic areas cranial and medial to the left kidney in an abdominal ultrasound. The cystic structure was drained with a needle and then with a laparoscopic suction device. The omentum was grasped with the forceps, and part of it was packed into the cystic structure with fixed margins. Then, partial cystectomy was performed with a vessel sealer-divider device. Abdominal ultrasound was performed 6 months after the surgery. There were no signs of previous pancreatic cysts. However, multiple small, newly developed cysts were found in the pancreas [58].

When surgical treatment is required for a macroscopically changed pancreas, omentalization is a great method. It can shorten recovery time and create a protective layer to help heal. However, the literature about pancreas omentalization is limited, and more research on patients would be indicated [51].

## 4. Omentum in Abscess Healing

Abscesses form when purulent material accumulates within the body, either septic or sterile. Causes of intra-abdominal abscesses include various factors, such as migrating foreign bodies, neoplasia, parasitic infections, and bacterial and fungal infections. Primary diseases affecting the retroperitoneum are rare, with reported occurrences including neoplastic masses, retroperitonitis, pneumoretroperitoneum, and non-neoplastic space-occupying lesions [67]. Retroperitonitis often arises from migrating foreign bodies and can lead to the formation of fistulous tracts. Diagnostic methods used to investigate fistulous tracts include radiology, contrast radiology, ultrasound, CT scans, and MR examinations [68,69].

The sublumbar muscles, positioned beneath the caudal thoracic and lumbar vertebrae, consist of layers such as the psoas minor, psoas major, iliacus, and quadratus lumborum muscle [8]. Adjacent to these muscles lie retroperitoneal structures, including the aorta, caudal vena cava, sublumbar lymph nodes, kidneys, ureters, adrenal glands, cisterna chyli, and neural structures. While primary diseases in this area are rare, neoplasia and abscesses have been documented [67]. Sublumbar abscessation, characterized by purulent collections within these muscles, often presents with discharging sinuses. Frendin et al.‘s [70] study on hunting dogs revealed plant material in abscesses, suggesting migration from the lungs through the diaphragm. The proximity of the diaphragm to the psoas muscles facilitates this migration. The predominance of Spaniel breeds in this condition aligns with previous reports, indicating a predisposition likely linked to hunting activities [70]. Woodbridge and Martinoli describe using of omentum in the treatment of sublumbar abscessation in 10 dogs. The medical files of dogs treated at Dick White Referrals, Cambridge, from January 2005 to December 2011 for managing sublumbar abscesses, possibly due to migrating plant material, were reviewed. Ten dogs underwent this treatment, including various breeds like Springer Spaniels and Labradors. Most dogs showed signs of lethargy and abdominal pain and had received prior antibiotic therapy. Procedures involved midline coeliotomy and exploration, including retraction of the duodenum or colon and penetration in the lumbar region due to access to the abscess cavity. The abscess was debrided, and the next step was the omentalization of the abscess cavity. Dogs were monitored post-surgery, and long-term follow-ups were conducted. All had abscesses in the psoas muscles, sometimes extending to nearby areas. Surgical complications were minimal, with only one dog experiencing aorta tear, which was successfully repaired. No postoperative issues were recorded, and most dogs fully recovered within a median of 1.5 years, except one that died due to unrelated trauma 18 months later [56]. In this study, surgical intervention involving exploration, debridement, and omentalization proved effective, even without identifying foreign bodies. This study suggests considering omentalization after exploration and debridement, especially when foreign material is undetected. In summary, surgical exploration with subsequent debridement, omentalization, and postoperative antibiotic therapy consistently yields successful outcomes for dogs with sublumbar abscessation.

**Figure 2:**
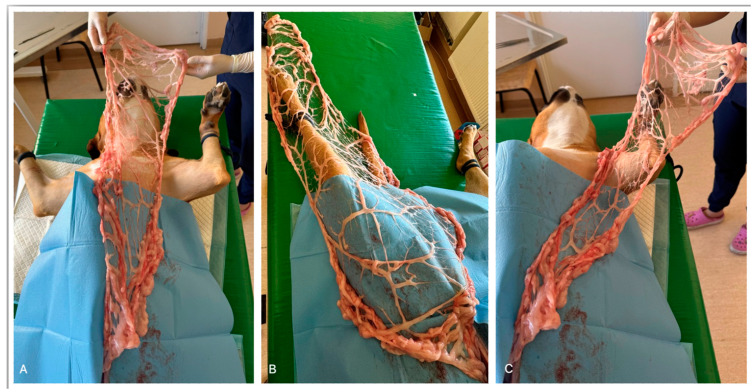
Distribution of omentum to the (**A**) head, (**B**) hind limb, and (**C**) forelimb. Examination on cadaver.

Mediastinal masses are commonly found in dogs, though those affecting the caudal mediastinum are less frequent [55]. Various causes of caudal mediastinal masses include neoplasia, diaphragmatic hernias, hematomas, and abscesses, the latter often resulting from esophageal perforation or foreign object migration [71,72,73]. While medical treatment can sometimes effectively manage pyothorax [74,75], surgery is advised if structural or granular lesions are detected, as the latter may suggest Actinomyces infection [76,77]. Drainage is considered a crucial therapeutic measure for abscesses [78], with omentum being particularly effective due to its extensive surface area and dense lymphoid tissue, facilitating efficient lymphatic drainage [9,79]. One advantageous characteristic of the omentum is its capacity to extend and enhance the effective coverage of the graft through lengthening (Figure 2) [80]. Additionally, the omentum is a source of macrophages, lymphocytes, and mast cells due to numerous lymphoid aggregates [13]. Nevertheless, there remains some debate surrounding omentalization due to the potential association of certain pathogens like Actinomyces and Nocardia with neoplasia [81,82]. Franklin et al. [82] described a case of a 2-year-old German Shorthaired Pointer with a mediastinal abscess, which was diagnosed with an x-ray and ultrasound examination. Surgical intervention was performed, revealing a large multiloculated pus-filled mass in the caudal thorax with adhesions to the diaphragm. The mass was excised, and the cavity was thoroughly irrigated. Positive ventilation was necessary to resolve lung compression caused by the mass. Omentalization was performed by advancing a pedicle flap of omentum into the thoracic cavity through a diaphragmatic defect, effectively filling and occluding the abscess cavity. Serous fluid drainage gradually decreased, and the thoracostomy tube was removed after 64 h. Cytological and culture analyses of abscess contents revealed bacterial infection, with histopathological analysis indicating granulation tissue consistent with an abscess. Follow-up radiography showed a reduction in abscess size, with no postoperative complications noted during suture removal or 3-month telephone follow-up. In this instance, the omentum was elongated and advanced into the thoracic cavity without tension by releasing the dorsal leaf of the omentum from its short epiploic vessels and disconnecting vascular connections with the spleen and pancreas [83].

## 5. Omentum in the Treatment of Cysts

Solitary cysts of the biliary system outside the liver are rare occurrences in cats and dogs [84]. Most cystic conditions are multifocal and linked to polycystic disease, typically affecting the kidneys or liver [85]. Conversely, biliary cystadenomas are often solitary but are usually situated within the liver parenchyma [86,87]. These structures are categorized into five types based on their anatomical location, with type 1—cystic or fusiform dilation of the common bile duct—being the most prevalent. Type 5 lesions, known as Caroli’s disease, involve secular dilations of the intrahepatic bile ducts and have been observed in a litter of Golden Retriever puppies with concurrent renal cysts. Type 1 cystic dilations of the bile ducts, termed choledochal cysts, typically contain bilious fluid rich in pancreatic enzymes [88]. Best et al. [83] described the case of a 10 cm choledochal cyst in the cat. After celiotomy, a sizable fluid-filled structure was identified in the mid-cranial abdomen, positioned adjacent to the caudal margin of the liver and connected to the common bile duct. Upon incision of the cyst, suction was employed to evacuate its contents, revealing a colorless, slightly cloudy fluid. Additionally, a small amount of more concentrated, bile-like fluid was found in the dependent portion of the cyst. Given the origin of the cyst from the common bile duct, masse resection was deemed impractical, leading to a subtotal resection of the cyst wall. Following the closure of the stomas at both ends using polypropylene purse-string sutures, omentalization of the structure was performed. The original cystostomy incision was partially sealed, and the residual cavity was filled with an omental pedicle. The patient experienced a rapid recovery and fully regained fitness within a short period [84].

Synovial cysts are characterized by a lining of synovial epithelium and are filled with synovial fluid [54]. The precise cause of these cysts remains unknown, but they are believed to form when the joint becomes distended, causing the synovial membrane to protrude through the joint capsule [89]. Synovial cysts in cats are rare, with eight out of nine reported cases involving the elbow joint and one case originating from an interphalangeal joint in a non-weight-bearing digit of a front limb [89,90,91,92]. Nearly all cysts were linked to degenerative joint disease (DJD) in the affected joint [93]. Treatments like simple resection or aspiration drainage frequently resulted in cyst recurrence within 2 weeks to 7 months when DJD was present [89,90,92,93]. Due to the drainage capacity of the omentum [47,94,95], a case of surgical treatment of a synovial cyst in a cat was described in 2015. A 13-year-old spayed female European domestic shorthair cat presented with left forelimb lameness lasting several months. The owner noticed a progressively enlarging, non-painful swelling on the inner left shoulder over several weeks. Physical examination was normal except for severe lameness in the left front limb (grade 4/5) and a soft, non-painful mass on the medial left humerus, extending from the shoulder joint to the distal humerus, not attached to the thoracic wall. Radiographs showed glenohumeral osteoarthritis with osteophytes and humeral head sclerosis, as well as a soft tissue density mass from the shoulder joint to the distal humerus. Aspiration of the mass yielded slightly yellow, transparent fluid with a tenacious string, indicating protein-rich inflamed synovial fluid with numerous pleomorphic cells and few macrophages [96]. The cat was placed in dorsal recumbency during the surgery, and a 12 cm craniomedial skin incision was made over the mass parallel to the humerus. The superficial pectoral muscles were separated, and the deep pectoral, deltoid, triceps, and biceps brachii muscles were dissected away from the mass. The thin, transparent wall of the mass was carefully separated from the surrounding tissues. Proximally, the mass wall was attached to the caudomedial aspect of the glenohumeral joint capsule. Upon opening the mass, it was discovered that its wall was continuous with the joint capsule. The mass wall was cut, creating an open connection with the joint. An osteophyte pointing toward the joint capsule defect was smoothed out using bone rongeurs. The joint capsule was closed in a continuous pattern. The subcutis and skin were closed over a 4 mm Penrose drain, which was placed proximomedially to the glenohumeral joint and exited medially 1 cm distal to the incision. No bandage was applied. Three weeks later, the owner noticed a recurrence of a soft, non-painful swelling that gradually increased in size. Examination revealed a fluctuating mass, and the cat had grade 1 lameness. Fine needle aspiration confirmed the synovial cyst recurrence, and the owner chose to proceed with another surgical intervention. During the second operation, the cyst wall was more firmly attached to the surrounding tissues during this surgery and was removed. The connection between the cyst and the shoulder joint was confirmed again. The joint capsule was closed as before. The greater omentum was harvested through a small left para costal mini-laparotomy and mobilized cranially through a subcutaneous tunnel to the axillary region. The omental pedicle was sutured to the joint capsule and surrounding tissues with monofilament sutures. The paracostal incision was partially closed, leaving enough space for the omentum to pass through without compromising its blood supply. Both incision sites were closed routinely. The cat recovered well with occasional grade 1 lameness. No recurrence was reported for three months, and clinical evaluation 14 weeks post-surgery showed no recurrence [96].

Perinephric pseudocyst formation involves fluid accumulation around the kidneys and is most seen in humans and cats. Arnold [97] reviewed 43 human cases, attributing 90 percent to trauma, either blunt or surgical, leading to urine leakage from the renal structures. From 1963 to 1998, thirteen cases in cats and two in dogs were documented [97,98,99,100,101,102,103,104,105,106,107,108,109,110,111,112]. Treatment options for perinephric pseudocysts include nephrectomy, cyst resection, and percutaneous needle aspiration [109,110,111,112]. Nephrectomy can exacerbate chronic renal failure in the remaining kidney [112], while needle drainage provides only temporary relief as fluid reaccumulates, sometimes within two days [102,108,112]. Treatments involve extensive resection of the cyst wall to prevent recurrence [97,98,99,102,112]. Incomplete cyst wall removal can lead to new pseudocyst formation [101]. Persistent fluid production with peritoneal reabsorption is suggested in perinephric pseudocyst cases [97,99,108]. In a case by Rishniw et al., [108] ascites developed after capsule resection, resolving only after nephrectomy. Inns (1997) reported using the omentum to aid cyst wall resection [101]. In 2000, Hill described the use of omentum for the surgical treatment of perinephric pseudocysts in cats. An 11-year-old male neutered domestic shorthair cat underwent X-ray and ultrasound tests, revealing a perinephric pseudocyst. During surgery, two large cysts were found in the upper abdomen and nearby kidneys. The cyst walls were removed with a 2 cm margin around each kidney’s hilum. Both kidneys appeared similar, with irregular surfaces, prominent blood vessels, and a grey-yellow color. The greater omentum was used to cover both kidneys without tension, and no omental pedicle extension was needed. The omentum was sutured around the cysts using a 4/0 polydioxanone thread. The abdomen was flushed with lactated Ringer’s solution and closed routinely. Seven months later, clinical and ultrasound exams showed no fluid buildup around the kidneys, and the health of the cat was good. The omentum was spread over the kidney and the floor of the abdomen to avoid blood vessel damage and maximize drainage. Direct contact with the renal parenchyma may have facilitated new capillary and lymph vessel growth, improved drainage, and reduced the risk of renal ischemia [113]. 

## 6. Omentum in the Treatment of Prostatic Diseases

As a well-blood-supplied structure, omentum provides good angiogenesis and absorbs bacteria and contaminating materials. It is valuable in many surgical treatment methods [114,115,116]. Thanks to those properties, ometalization can also be used for prostate diseases in dogs. Non-castrated male dogs can be exposed to many prostatic pathologies, such as prostatitis, benign prostatic hyperplasia (BPH), and less frequent prostatic cysts, abscesses, or neoplasia [48,49,117,118,119]. In many cases, castration of intact dogs, together with pharmacological therapy, is the recommended method of prostate disease treatment [120,121].

Although prostatic cysts are not common, they can become infected in almost 42% of cases [48,49]. We can recognize parenchymal prostatic cysts, with an accumulation of secretions from the prostatic gland caused by BPH. They can be connected to the urethra. Extraparenchymal prostatic cysts are the second type, connected to the prostatic capsule, but do not involve prostatic parenchyma [49,122,123].

Drainage and omentalization are highly effective surgical methods in prostatic lesions. The omentum is placed in lesions or lacerations after their debridement, ensuring rapid tissue healing. It is passed by the capsulectomy site and around the urethra to provide the correct blood supply [49,94,120].

In omentalization, the placement of the omentum can impact the recurrence of abscesses or cysts. If a urethral laceration is present, the omentum is placed along the defect and attached to the prostatic capsules with proper sutures. Usually, a urinary catheter is placed for better healing [94,120]

Omentalization of prostatic cavities is a better treatment option than ventral drainage, marsupialization, or prostatectomy because postoperative care is faster, less complicated, and less time-consuming [94,124,125]. As the only treatment method, ventral drainage has been proven to be a good method, but prostatic lesions can have adhesion to the urethra or bladder neck. In the long-term outcome, they can cause urinary retention and incontinence [126,127,128]. Among the side effects of marsupialization are chronic urinary tract infections, abscessation, or persistent stoma discharge [129]. On the other hand, partial resection has minimal effect on incontinence but does not stop fluid secretion, and cysts can redevelop [93].

White and Williams described intracapsular prostatic omentalization in the treatment of prostatic abscesses in dogs. During celiotomy, the prostate gland was exposed with laparotomy sponges. Bilateral incisions in the lateral part of the prostate allowed the removal of the pus by suction. Around the prostatic urethra, a Penrose drain was placed to irrigate abscess cavities. The omentum was passed by the capsulectomy wound and prostatic urethra and anchored with a mattress suture. Of the 20 dogs that participated in that study, 19 of them reported no abscess recurrence for 12 months of postoperation follow-up [95]. More recent studies are available that describe using the intracapsular omentalization method, with similar, successful outcomes in 44 treated dogs [49]. In the case of 11 dogs, intraparenchymal prostatic cysts were diagnosed, they were drained, and ometalization was performed during surgery. In extraparenchymal cysts, partial resection and omentalization were performed in 26 of 33 dogs in this group, where complete cyst removal was in 7 of 33 dogs. The median follow-up was 528 days. Prostatic cyst recurrence was observed in 2 dogs with extraparenchymal cysts after partial cyst removal and omentalization. There was no recurrence in dogs where extraparenchymal cysts were completely removed [49].

One of the most common complications in the mentioned studies was urinary incontinence [49,93]. However, considering that prostatic defects did not recur in most patients, omentalization used to treat prostatic cavities is a method that gives satisfying results. 

## 7. Omentum in Bone Healing

Due to the presence of ADSCs in the omentum, this structure may be active in treating fractures in dogs and cats [17]. The perspective on ADSCs has shifted, considering recent research, which has highlighted a variety of bioactive substances released by ADSCs that may be crucial for bone fracture healing [30]. A glycosylphosphatidylinositol (GPI) anchor holds the ecto-5′-nucleotidase CD73 to the outer plasma membrane. CD73 is an adhesion molecule and signal that assists cell anchoring [25,130]. Research suggests that the expression of CD73 may be correlated with the donor’s age; younger donors showed higher expression, whereas older donors showed lower expression [131]. According to recent research, CD73 regulates the osteogenic differentiation of ADSCs, and its absence lowers the amount of bone minerals in the bone [131,132]. The Wnt/β-catenin signaling system, a crucial mechanism in osteogenic activation, controls the expression of CD73. Growth factors and cytokines, including TGF-β, TNF-α, and IL-1β, which are present in the early stages of bone repair, also control the production of this marker [130]. The membrane protein CD90, which is associated with glycosylphosphatidylinositol (GPI), is expressed on the surfaces of fibroblasts, peripheral T cells, epithelial cells, neurons, and thymocytes in addition to hematopoietic stem cells. Nonetheless, some research has shown that the differentiation state of osteoblast lineage cells tends to influence CD90 expression. As calcified nodules form, CD90 expression seems to increase in proliferating cells and decrease as the cells go through the matrix maturation and mineralization stages [133,134]. In addition, the RUNX2 marker was isolated from ADSCs dogs from omentum [27,28], which is a master transcription factor for osteogenesis [135]. Moreover, due to the ability to produce factors directly involved in osteogenesis, omental cells can also produce factors stimulating angiogenesis, e.g., VEGF, which accelerates the bone healing process [136].

There are few reports on using omentum in bone healing in the current literature. In 2009, Saifzadeh et al. [137] reported the potentiality of the autogenous nonvascularized free omentum graft to stimulate bone healing in an experimental hypertrophic nonunion model on twelve dogs. A verified model of nonunion was utilized through a standardized transverse mid-diaphyseal radial osteotomy while keeping the ulna intact and leaving the operated limb uncased. In both groups of dogs, access to the abdominal cavity was gained through a 3 cm incision along the ventral midline, positioned halfway between the umbilicus and pelvic inlet. The greater omentum was then found and brought out from the abdominal cavity. Using two catgut ligatures, a 30 × 30 mm^2^ section of the larger omentum was isolated and separated from the remainder of the omental pedicle. The omental patch was cautiously wrapped in laparotomy pads moistened with saline. The removed portion of the omentum was placed over the osteotomy gap in the experimental group as a free graft and fastened with tack sutures using 3/0 polyglycolate. No internal or external fixation devices were applied after surgery. After a 4-month follow-up period, both radiological and histological assessments of the cohort treated with a free transplant of the greater omentum exhibited full union. Conversely, the control group showed no signs of a union. The evaluation of radiological and histological scores corroborated that osteotomies treated with a free transplant of autogenous greater omentum had successfully united, whereas those in the control group had not [137].

McAlinden et al. [51] described a case report of using omentalization as supplementary treatment of an infected femoral nonunion fracture in a three-year-old, 16 kg body weight Border Collie. The dog had experienced a closed, comminuted, mid-shaft right femoral fracture. Initially, a type Ia external skeletal fixator was applied, but it failed six weeks postoperatively due to premature pin loosening and loss of stability. Subsequently, a plate-and-rod construct was applied in a second surgery using an intramedullary pin and dynamic compression plate (DCP), which also failed. A ventral midline coeliotomy was performed eight months after the initial surgery [52]. A two-stage omental pedicle extension technique was conducted, according to Ross and Pardo [80]. A 3 cm paramedian stab incision was made in the right abdominal wall, 4 cm lateral to the prepuce, through which the omentum was exteriorized and tunneled subcutaneously to the cranial aspect of the right thigh [80]. The femur was exposed using a standard lateral approach described by Piermattei and Johnson [138]. The omental pedicle was brought cranially and laterally to the sartorius muscle during the approach. The fracture was found to be unstable and surrounded by abundant serosanguinous fluid. Necrotic soft tissue was carefully debrided without disturbing the fracture fragments. The intramedullary pin and the heads of the fractured screws in the distal fragment were removed, and the omental pedicle was gently packed into the fracture gap, ensuring it was not under tension. Surgical wound closure followed a routine procedure, with the exception that the fascia lata was incompletely closed in the proximal half of the wound to prevent the vascular supply of the omental pedicle [138]. The patient underwent re-examination at four-week intervals over a span of 16 weeks. Sixteen weeks postoperatively, the dog displayed only mild residual lameness, and both clinical and radiographical evidence confirmed bone union [52].

In 2015, Baltzer et al. [139] published a retrospective clinical study that reviewed the clinical outcome in 25 dogs with a body weight of <6 kg with mid to distal diaphyseal fractures of the radius and ulna treated with open reduction bone plate fixation with and without the use of a free autogenous greater omental graft. This study used insulated excised omentum to treat thirteen fractures in 11 dogs. All dogs in the omental group underwent a ventral midline celiotomy, positioned 1 cm caudal to the umbilicus and measuring 2 cm in length, in addition to the dorsal-medial surgical approach to the fractured radius. A 2–3 cm^3^ section of greater omentum was isolated, ligated, and transected using a single wraparound ligature of absorbable monofilament suture. The omental graft was placed over the bone plate and wrapped around the radius and ulna, ensuring contact with the bone surfaces medially and laterally. Special attention was given to ensuring omentum-bone contact on the radius, both proximally and distally to the bone plate, as well as on the ulna medially and laterally. Both the celiotomy and antebrachial incisions were closed using standard procedures [139]. Closing the fracture site incision was subjectively considered more challenging with the omentum grafts placed over the bone plate and radius compared with the closure in the non-omentum group. However, complete closure was successfully achieved in all cases. The median time to complete radiographic healing in the omental group dogs was 70.0 days compared with 106 days in the non-omental group [139]. 

The latest report by Ree et al. [140] aimed to determine the rate of radiographic healing, complications, vascularization, and bone density following the repair of radial and ulnar fractures in dogs weighing less than 6 kg. These dogs were treated with either an autogenous cancellous bone graft or a free autologous omentum graft [140]. For the omentum group dogs, a 3 cm-long ventral midline celiotomy was performed. A 2–3 cm^2^ portion of the greater omentum was ligated, transected, and placed over the bone plate, ensuring contact with the radius and ulna both proximally, distally, and medially, as previously described [139]. For the bone graft group, approximately 1–4 mm^3^ of cancellous bone was harvested from a 2.5-mm drilled hole in the proximal humerus and immediately placed around the fracture site [140]. Radiographic healing occurred earlier in bones treated with omentum grafts (median of 9 weeks) than those treated with bone grafts (12 weeks). However, cortical bone density, as measured with CT of the distal ulna, was higher in bones that received bone graft augmentation than those with omentum grafts [140].

The remarkable capability of the omentum in tissue revascularization has been extensively documented in the medical literature. Numerous studies have highlighted the angiogenic potential of omental fat in this regard [141,142]. Neovascularization empowers the omentum to offer vascular support and facilitate function and healing in ischemic or inflamed tissue [143,144].

Omental grafting has been successfully used in humans to heal fractures and soft tissue wounds [145]. Omentum-induced angiogenesis and fracture healing are believed to occur through VEGF-mediated neovascularization, callus formation, and mineralization [137,146]. Effective bone healing after a fracture depends on forming new capillaries from existing blood vessels; without this angiogenesis, delayed unions and nonunions can result [147,148]. Vascular endothelial growth factor (VEGF) is released from activated omental adipocytes and stromal cells at a rate 10–20 times higher than primary mesangial or glomerular epithelial cells [149]. VEGF has been found to stimulate bone repair by promoting blood vessel formation and the maturation and ossification of new bone callus in mouse and rabbit fractures [150,151]. In addition to releasing VEGF, the activated omentum also releases basic fibroblast growth factor and transforming growth factor-β, both of which are reported to have positive effects on bone repair [152,153,154].

## 8. Omentum in Wound Healing

Wound healing is a long-term process in veterinary medicine [47]. The use of omentum has been documented to improve the healing of chronic wounds in cats, including those in the axillary region [155,156]. In 1998, Lascelles described the use of omentum for difficult-to-heal axillary wounds in 10 cats. These cats with chronic, non-healing axillary wounds were treated at two veterinary hospitals between May 1992 and March 1997. These cats had previously undergone various unsuccessful treatments, including debridement and primary closure, supplemented with antibiotics. At the hospitals, they underwent further diagnostic tests and surgery to debride and omentalize the wounds for primary closure. General anesthesia was administered, and the wounds were meticulously prepared and debrided. The omentum was surgically mobilized and used to aid wound closure. Some cats required additional wound repairs due to complications. Outcomes were assessed based on the healing of the axillary wounds within two months post-surgery. Most cats healed successfully, although a few experienced complications such as wound dehiscence, hernias, or anorexia. Overall, eight cats showed good recovery, while two had poor or failed outcomes. Despite some issues, limb function was generally unaffected, and no long-term lameness was observed [47].

The study found that using the omentum to treat chronic non-healing axillary wounds in cats is effective, likely due to the blood supply, drainage, and reduction of dead space it provides. On average, seven of ten cats had wounds that healed within 24 days. Chronic conditions and mechanical factors like tension and movement may hinder wound healing, but thorough debridement and omentalization can improve outcomes [47,157]. Previous attempts at wound repair using various techniques had failed, but the omentum’s properties, such as aiding revascularization and reducing dead space, made it suitable for this purpose [53,158]. Despite early wound dehiscence in some cases, the presence of the omental graft facilitated healing. Reconstructive procedures to relieve tension were not used, and spica bandages seemed to hinder rather than help to heal due to suboptimal tension [159,160,161]. This study noted incisional hernias but did not cause long-term problems. Some cats experienced excessive discharge, possibly from partial omental fat necrosis, but this did not prevent healing. No long-term gastrointestinal issues were reported, suggesting that the orientation of the gastrointestinal tract in quadrupeds might prevent complications [47]. Overall, the study supports the use of omentalization for managing chronic wounds in cats despite some postoperative challenges. 

Combining an omental flap with a skin flap significantly improved the outcome. Lascelles and White introduced the thoracodorsal axial pattern skin flap. In their study, only two out of 10 cats needed additional surgery. This flap technique utilizes the cutaneous branch of the thoracodorsal artery, which emerges just behind the shoulder angle and extends dorsally [162]. Gray, in turn, described the use of an omocervical axial pattern flap in the treatment of difficult-to-heal axillary wounds in cats. A mature, neutered male domestic shorthair cat had a large, chronic wound in the right axillary area. A tissue culture revealed *Staphylococcus aureus*, and histology indicated chronic pyogranulomatous dermatitis. Under general anesthesia, an omental flap was mobilized through a midline coeliotomy and redirected to the wound site. Scar tissue was excised, and an omocervical axial pattern skin flap was used to cover the wound. The flap was sutured in place, and a closed suction drain was installed for three days. Postoperatively, a mild discharge and a small necrotic area were noted, which healed with additional antibiotics. The wound showed good progress, completely healing by the six-week follow-up. 

## 9. Conclusions

The omentum is very important in soft surgery and orthopedics, yet many surgeons overlook it. Modern veterinary literature needs studies that delve into its functioning and potential applications. Until now, there have been limited reports on the use of omentum in orthopedics, presenting a wide opportunity for researchers to explore. In the scientific reports mentioned earlier, utilizing omentum showed beneficial therapeutic outcomes. There are no documented negative effects of using omentum in veterinary medicine.

Most applications of this structure are predominantly observed in reports from soft surgery. This is primarily attributed to its drainage capabilities, the presence of inflammatory cells, and its capacity to promote angiogenesis. It is still an undiscovered organ. Exploring the cellular structure of the omentum, especially the existence of stem cells that aid in tissue reconstruction, is worth investigating. 

## Data Availability

No new data were created or analyzed in this study.

## References

[1] Barone R. (2009). Appareil Digestif et Appareil Respiratoire. Anatomie Comparee des Mammiferes Domestiques. Tome 3, Splanchnologie 1.

[2] Doom M., de Rooster H., van Bergen T., Gielen I., Kromhout K., Simoens P., Cornillie P. (2016). Morphology of the Canine Omentum Part 1: Arterial Landmarks that Define the Omentum. Anat. Histol. Embryol..

[3] McGeady T.A., Quinn P.J., FitzPatrick E.S., Ryan M.T. (2006). Coelomic Cavities. Veterinary Embryology.

[4] Doom M., de Rooster H., van Bergen T., Gielen I., Kromhout K., Simoens P., Cornillie P. (2016). Morphology of the Canine Omentum Part 2: The Omental Bursa and its Compartments Materialized and Explored by a Novel Technique. Anat. Histol. Embryol..

[5] Liu M., Silva-Sanchez A., Randall T.D., Meza-Perez S. (2021). Specialized immune responses in the peritoneal cavity and omentum. J. Leukoc. Biol..

[6] Budras K.D., Budras K.-D. (2007). Abdominal Cavity. Anatomy of the Dog.

[7] Zietzschmann O. (1939). Das Mesogastrium dorsale des Hundes mit einer schematischen Darstellung seiner Blatter. Morphol. Jahrb..

[8] Evans H., De Lahunta A., Evans H.E. (2013). The digestive apparatus and abdomen. Miller’s Anatomy of the Dog.

[9] Hosgood G. (1990). The omentum—The forgotten organ—Physiology and potential surgical applications in dogs and cats. Compend. Contin. Educ. Vet..

[10] Michailova K.N., Usunoff K.G. (2004). The milky spots of the peritoneum and pleura: Structure, development and pathology. Biomed. Rev..

[11] Huyghe S., de Rooster H., Doom M., Van den Broeck W. (2016). The Microscopic Structure of the Omentum in Healthy Dogs: The Mystery Unravelle. Anat. Histol. Embryol..

[12] Ryan G.B., Grobety J., Majno G. (1971). Postoperative peritoneal adhesions. A study of the mechanisms. Am. J. Pathol..

[13] Shimotsuma M.J., Shields J., Simpson-Morgan M.W., Sakuyama A., Shirasu M., Hagiwara A., Takahashi T. (1993). Morpho-physiological function and role of omental milky spots as omentum-associated lymphoid tissue (OALT) in the peritoneal cavity. Lymphology.

[14] Wilkosz S.G., Ireland N., Khwaja N., Walker M., Butt R., de Giorgio-Miller A., Herrick S.E. (2005). A comparative study of the structure of human and murine greater omentum. Anat. Embryol..

[15] Krist L.F., Eestermans I.L., Steenbergen J.J., Hoefsmit E.C., Cuesta M.A., Meyer S., Beelen R.H. (1995). Cellular composition of milky spots in the human greater omentum: An immunochemical and ultrastructural study. Anat. Rec..

[16] Shimotsuma M., Kawata M., Hagiwara A., Takahashi T. (1989). Milky spots in the human greater omentum. Macroscopic and histological identification. Acta Anat..

[17] Ferreira-Baptista C., Ferreira R., Fernandes M.H., Gomes P.S., Colaco B. (2023). Influence of the Anatomical Site on Adipose Tissue-Derived Stromal Cells’ Biological Profile and Osteogenic Potential in Companion Animals. Vet. Sci..

[18] Merlo B., Iacono E. (2023). Beyond Canine Adipose Tissue-Derived Mesenchymal Stem/Stromal Cells Transplantation: An Update on Their Secretome Characterization and Applications. Animals.

[19] Bach F.S., Rebelatto C.L.K., Fracaro L., Senegaglia A.C., Fragoso F.Y.I., Daga D.R., Brofman P.R.S., Pimpão C.T., Engracia Filho J.R., Montiani-Ferreira F. (2019). Comparison of the Efficacy of Surgical Decompression Alone and Combined with Canine Adipose Tissue-Derived Stem Cell Transplantation in Dogs with Acute Thoracolumbar Disk Disease and Spinal Cord Injury. Front. Vet. Sci..

[20] Bahamondes F., Flores E., Cattaneo G., Bruna F., Conget P. (2017). Omental adipose tissue is a more suitable source of canine Mesenchymal stem cells. BMC Vet. Res..

[21] Chae H.-K., Song W.-J., Ahn J.-O., Li Q., Lee B.-Y., Kweon K., Park S.-C., Youn H.-Y. (2017). Immunomodulatory effects of soluble factors secreted by feline adipose tissue-derived mesenchymal stem cells. Vet. Immunol. Immunopathol..

[22] De Cesaris V., Grolli S., Bresciani C., Conti V., Basini G., Parmigiani E., Bigliardi E. (2017). Isolation, proliferation and characterization of endometrial canine stem cells. Reprod. Domest. Anim..

[23] Enciso N., Ostronoff L.L.K., Mejías G., León L.G., Fermín M.L., Merino E., Fragio C., Avedillo L., Tejero C. (2018). Stem cell factor supports migration in canine mesenchymal stem cells. Vet. Res. Commun..

[24] Krueger E., Magri L.M.S., Botelho A.S., Bach F.S., Rebellato C.L.K., Fracaro L., Fragoso F.Y.I., Villanova J.A., Brofman P.R.S., Popović-Maneski L. (2019). Effects of low-intensity electrical stimulation and adipose derived stem cells transplantation on the time-domain analysis-based electromyographic signals in dogs with SCI. Neurosci. Lett..

[25] Rashid U., Yousaf A., Yaqoob M., Saba E., Moaeen-ud-Din M., Waseem S., Becker S.K., Sponder G., Aschenbach J.R., Sandhu M.A. (2021). Characterization and differentiation potential of mesenchymal stem cells isolated from multiple canine adipose tissue sources. BMC Vet. Res..

[26] Requicha J.F., Viegas C.A., Albuquerque C.M., Azevedo J.M., Reis R.L., Gomes M.E. (2012). Effect of Anatomical Origin and Cell Passage Number on the Stemness and Osteogenic Differentiation Potential of Canine Adipose-Derived Stem Cells. Stem Cell Rev. Rep..

[27] Ferreira-Baptista C., Queirós A., Ferreira R., Fernandes M.H., Gomes P.S., Colaço B. (2023). Retinoic acid induces the osteogenic differentiation of cat adipose tissue-derived stromal cells from distinct anatomical sites. J. Anat..

[28] Linkova D.D., Rubtsova Y.P., Egorikhina M.N. (2022). Cryostorage of Mesenchymal Stem Cells and Biomedical Cell-Based Products. Cells.

[29] Vizoso F.J., Eiro N., Cid S., Schneider J., Perez-Fernandez R. (2017). Mesenchymal Stem Cell Secretome: Toward Cell-Free Therapeutic Strategies in Regenerative Medicine. Int. J. Mol. Sci..

[30] Mocchi M., Dotti S., Del Bue M., Villa R., Bari E., Perteghella S., Torre M.L., Grolli S. (2020). Veterinary Regenerative Medicine for Musculoskeletal Disorders: Can Mesenchymal Stem/Stromal Cells and Their Secretome Be the New Frontier?. Cells.

[31] Yaneselli K.M., Kuhl C.P., Terraciano P.B., de Oliveira F.S., Pizzato S.B., Pazza K., Magrisso A.B., Torman V., Rial A., Moreno M. (2018). Comparison of the characteristics of canine adipose tissue-derived mesenchymal stem cells extracted from different sites and at different passage numbers. J. Vet. Sci..

[32] Buote N.J. (2022). Laparoscopic adipose-derived stem cell harvest technique with bipolar sealing device: Outcome in 12 dogs. Vet. Med. Sci..

[33] Fujimoto Y., Yokozeki T., Yokoyama A., Tabata Y. (2020). Basic fibroblast growth factor enhances proliferation and hepatocyte growth factor expression of feline mesenchymal stem cells. Regen. Ther..

[34] Kono S., Kazama T., Kano K., Harada K., Uechi M., Matsumoto T. (2014). Phenotypic and functional properties of feline dedifferentiated fat cells and adipose-derived stem cells. Vet. J..

[35] Li D., Luo H., Ruan H., Chen Z., Chen S., Wang B., Xie Y. (2021). Isolation and identification of exosomes from feline plasma, urine and adipose-derived mesenchymal stem cells. BMC Vet. Res..

[36] Parys M., Nelson N., Koehl K., Miller R., Kaneene J.B., Kruger J.M., Yuzbasiyan-Gurkan V. (2016). Safety of Intraperitoneal Injection of Adipose Tissue-Derived Autologous Mesenchymal Stem Cells in Cats. J. Vet. Intern. Med..

[37] Quimby J.M., Webb T.L., Habenicht L.M., Dow S.W. (2013). Safety and efficacy of intravenous infusion of allogeneic cryopreserved mesenchymal stem cells for treatment of chronic kidney disease in cats: Results of three sequential pilot studies. Stem Cell Res. Ther..

[38] Villatoro A.J., Claros S., Fernández V., Alcoholado C., Fariñas F., Moreno A., Becerra J., Andrades J.A. (2018). Safety and efficacy of the mesenchymal stem cell in feline eosinophilic keratitis treatment. BMC Vet. Res..

[39] Villatoro A.J., Martín-Astorga M.D.C., Alcoholado C., Cárdenas C., Fariñas F., Becerra J., Visser R. (2021). Altered Proteomic Profile of Adipose Tissue-Derived Mesenchymal Stem Cell Exosomes from Cats with Severe Chronic Gingivostomatitis. Animals.

[40] Etzerodt A., Moulin M., Doktor T.K., Delfini M., Mossadegh-Keller N., Bajenoff M., Sieweke M.K., Moestrup S.K., Auphan-Anezin N., Lawrence T. (2020). Tissue-resident macrophages in omentum promote metastatic spread of ovarian cancer. J. Exp. Med..

[41] Schreiber S., Gehrckens A., Raedler A. (1995). Activation of the major omentum-associated lymphoid tissue in Crohn disease. Zentralbl. Chir..

[42] Jani K., Saxena A.K., Vaghasia R. (2006). Omental plugging for large-sized duodenal peptic perforations: A prospective Gene detection in peritonitis by DD RT-PCR 173 randomized study of 100 patients. South. Med. J..

[43] Azouz A., Razzaque M.S., El-Hallak M., Taguchi T. (2004). Immunoinflammatory responses and fibrogenesis. Med. Electron. Microsc..

[44] Hultman C.S., Carlson G.W., Losken A., Jones G., Culbertson J., Mackay G., Bostwick J., Jurkiewicz M.J. (2002). Utility of the omentum in the reconstruction of complex extraperitoneal wounds and defects. Ann. Surg..

[45] Ito K.C., Ferrigno C.R.A., Alves F.R. (2010). Maximum length of greater omentum pedicle flap through subcutaneous tunnel for long bones in dogs. Cienc. Rural..

[46] Lascelles B.D., Davison L., Dunning M., Bray J.P., White R.A. (1998). Use of omental pedicle grafts in the management of non-healing axillary wounds in 10 cats. J. Small Anim. Pract..

[47] Black M.G., Ling G.V., Nyland T.G., Baker T. (1998). Prevalence of Prostatic Cysts in Adult, Large-Breed Dogs. J. Am. Anim. Hosp. Assoc..

[48] Del Magno S., Pisani G., Dondi F., Cinti F., Morello E., Martano M., Foglia A., Giacobino D., Buracco P. (2021). Surgical treatment and outcome of sterile prostatic cysts in dogs. Vet. Surg..

[49] Jerram R.M., Warman C.G., Davies E.S.S., Robson M.C., Walker A.M. (2004). Successful treatment of a pancreatic pseudocyst by omentalisation in a dog. N. Z. Vet. J..

[50] Johnson M.D., Mann F.A. (2006). Treatment for pancreatic abscesses via omentalization with abdominal closure versus open peritoneal drainage in dogs: 15 Cases (1994–2004). J. Am. Vet. Med. Assoc..

[51] McAlinden A., Glyde M., McAllister H., Kirby B. (2009). Omentalisation as adjunctive treatment of an infected femoral nonunion fracture: A case report. Ir. Vet. J..

[52] Pavletic M.M., Sherding R.G. (1994). Surgery of the skin and management of wounds. The Cat: Diseases and Clinical Management.

[53] Steiner E., Steinbach L.S., Schnarkowski P., Tirman P.F., Genant H.K. (1996). Ganglia and cysts around joints. Radiol. Clin. N. Am..

[54] Thrall D.E., Thrall D.E. (2002). The mediastinum. Textbook of Veterinary Diagnostic Radiology.

[55] Woodbridge N., Martinoli S., Cherubini G.B., Caine A., Nelissen P., White R. (2014). Omentalisation in the treatment of sublumbar abscessation: Long-term outcome in 10 dogs. Vet. Rec..

[56] Branter E.M., Viviano K.R. (2010). Multiple recurrent pancreatic cysts with associated pancreatic inflammation and atrophy in a cat. J. Feline Med. Surg..

[57] Brückner M. (2019). Laparoscopic omentalization of a pancreatic cyst in a cat. J. Am. Vet. Med. Assoc..

[58] Coleman M.G., Robson M.C., Harvey C. (2005). Pancreatic cyst in a cat. N. Z. Vet. J..

[59] Hines B.L., Salisbury S.K., Jakovljevic S., DeNicola D.B. (1996). Pancreatic pseudocyst associated with chronic-active necrotizing pancreatitis in a cat. J. Am. Anim. Hosp. Assoc..

[60] Lee M., Kang J.H., Chang D., Na K.J., Yang M.P. (2015). Pancreatic abscess in a cat with diabetes mellitus. J. Am. Anim. Hosp. Assoc..

[61] Marchevsky A.M., Yovich J.C., Wyatt K.M. (2000). Pancreatic pseudocyst causing extrahepatic biliary obstruction in a dog. Aust. Vet. J..

[62] Sadler R.A., Fields E.L., Whittemore J.C. (2016). Attempted ultrasound-guided ethanol ablation of a suspected pancreatic pseudocyst in a dog. Can. Vet. J..

[63] Smith S.A., Biller D.S. (1998). Resolution of a Pancreatic Pseudocyst in a Dog Following Percutaneous Ultrasonographic-Guided Drainage. J. Am. Anim. Hosp. Assoc..

[64] VanEnkevort B.A., O’Brien R.T., Young K.M. (1999). Pancreatic pseudocysts in 4 dogs and 2 cats: Ultrasonographic and clinicopathologic findings. J. Vet. Intern. Med..

[65] Thompson L.J., Seshadri R., Raffe M.R. (2009). Characteristics and outcomes in surgical management of severe acute pancreatitis: 37 dogs (2001–2007). J. Vet. Emerg. Crit. Care.

[66] Tobias K.M., Johnston S.A., Kirby B.M., Cornell K. (2012). Peritoneum and retroperitoneum. Veterinary Surgery Small Animal.

[67] Lamb C.R., White R.N., Mcevoy F.J. (1994). Sinography in the investigation of draining tracts in small animals: Retrospective review of 25 cases. Vet. Surg..

[68] Slatter D., Kirby B.M. (2003). Peritoneum and peritoneal cavity. The Textbook of Small Animal Surgery.

[69] Frendin J., Funkquist B., Hansson K., Lonnemark M., Carlsten J. (1999). Diagnostic imaging of foreign body reactions in dogs with diffuse back pain. J. Small Anim. Pract..

[70] Frendin J. (1997). Pyogranulomatous pleuritis with empyema in hunting dogs. Zentralbl Vet. A.

[71] Lotti U., Niebauer G.W. (1992). Tracheobronchial foreign bodies of plant origin in 153 hunting dogs. Comp. Cont. Educ. Pract. Vet..

[72] Schultz R.M., Zwingenberger A. (2008). Radiographic, computed tomographic, and ultrasonographic findings with migrating intrathoracic grass awns in dogs and cats. Vet. Radiol. Ultrasound.

[73] Johnson M.S., Martin M.W.S. (2007). Successful medical treatment of 15 dogs with pyothorax. J. Small Anim. Pract..

[74] Piek C.J., Robben J.H. (2000). Pyothorax in nine dogs. Vet. Q..

[75] Rooney M.B., Monnet E. (2002). Medical and surgical treatment of pyothorax in dogs: 26 cases (1991–2001). J. Am. Vet. Med. Assoc..

[76] Scott J.A., Macintire D.K. (2003). Canine pyothorax: Clinical presentation, diagnosis, and treatment. Comp. Cont. Educ. Pract. Vet..

[77] Knight H.D., Hietala S.K., Jang S. (1980). Antibacterial treatment of abscesses. J. Am. Vet. Med. Assoc..

[78] Nylander G., Tjernberg B. (1969). The lymphatics of the greater omentum: An experimental study in the dog. Lymphology.

[79] Ross W.E., Pardo A.D. (1993). Evaluation of an omental pedicle extension technique in the dog. Vet. Surg..

[80] Brown J.R. (1973). Human actinomycosis: A study of 181 subjects. Hum. Pathol..

[81] Smego R.A., Foglia G. (1998). Actinomycosis. Clin. Infect. Dis..

[82] Franklin A.D., Fearnside S.M., Brain P.H. (2011). Omentalisation of a caudal mediastinal abscess in a dog. Aust. Vet. J..

[83] Best E.J., Bush D.J., Dye C. (2010). Suspected choledochal cyst in a domestic shorthair cat. J. Feline Med. Surg..

[84] Domanjko-Petric A., Cernec D., Cotman M. (2008). Polycystic kidney disease: A review and occurrence in Slovenia with comparison between ultrasound and genetic testing. J. Feline Med. Surg..

[85] Adler R., Wilson D.W. (1995). Biliary cystadenoma of cats: Review. Vet. Pathol..

[86] Van den Ingh T.S.G.A.M., Cullen J.M., Twedt D.C., Winkle T.V., Desmet V.J., Rothuizen J. (2006). Morphological classification of biliary disorders of the canine and feline liver. WSAVA Standards for Clinical and Histological Diagnosis of Canine and Feline Liver Diseases.

[87] Besner E., Paddock H.N., Nguyen L.T., Kay S.M. (2008). Choledochal Cyst: A Surgical Perspective. e-Med. Rev..

[88] Kligman K.C., Kim S.E., Winter M.D., Bacon N.J., Krellner H.L., Levy J.K. (2009). What is your diagnosis? Synovial cysts. J. Am. Vet. Med. Assoc..

[89] Prymak C., Goldschmidt M.H. (1991). Synovial cysts in five dogs and one cat. J. Am. Anim. Hosp. Assoc..

[90] Stead A.C., Else R.W., Stead M.C. (1995). Synovial cysts in cats. J. Small Anim. Pract..

[91] White J.D., Martin P., Hudson D., Clark A., Malik R. (2004). What is your diagnosis?. J. Feline Med. Surg..

[92] Hittmair K.M., Maedl I., Reifinger M., Mayrhofer E. (2010). Synovial cyst of the fifth digit in a cat. J. Feline Med. Surg..

[93] Bray J.P., White R.A.S., Williams J.M. (1997). Partial resection and omentalization: A new technique for management of prostatic retention cysts in dogs. Vet. Surg..

[94] White R.A., Williams J.M. (1995). Intracapsular prostatic omentalization: A new technique for management of prostatic abscesses in dogs. Vet. Surg..

[95] Stegan L., Van Goethem B., Beerden C., Grussendorf C., de Rooster H. (2012). Use of greater omentum in the surgical treatment of a synovial cyst in a cat. Tierarztl. Prax. Ausg. K Kleintiere Heimtiere.

[96] Arnold E.P. (1972). Pararenal pseudocyst. Br. J. Urol..

[97] Abdinoor D.J. (1980). Perinephric pseudocysts in a cat. J. Am. Anim. Hosp. Assoc..

[98] Carlson R.A., Badertscher R.N. (1993). Feline renal pseudocyst with metastatic carcinoma of the contralateral kidney. Feline Pract..

[99] Chastain C.B., Grier R.L. (1975). Bilateral retroperitoneal cysts in a cat. Feline Pract..

[100] Geel J.K. (1986). Perinephric extravasation of urine with pseudocyst formation in a cat. J. S. Afr. Vet. Assoc..

[101] Inns J.H. (1997). Treatment of perinephric pseudocysts by ornental drainage. Aust. Vet. Pract..

[102] Kirberger R.M., Jacobsen L.S. (1992). Perinephric pseudocysts in a cat. Aust. Vet. Pract..

[103] Kraft A.M., Kraft C.G. (1970). Renal capsular cyst in a domestic cat. Vet. Med. Small Anim. Clin..

[104] Lemire T.D., Reao W.K. (1998). Macroscopic and microscopic characterization of a urineferous perirenal pseudocyst in a domestic short hair cat. Vet. Pathol..

[105] Miles K.G., Jergens A.E. (1992). Unilateral perinephric pseudocyst of undetermined origin in a dog. Vet. Radiol. Ultrasound.

[106] Mitten R.A. (1978). Pararenal pseudocysts in a cat. Iowa State Univ. Vet..

[107] Moon M.L., Dallman M.A. (1991). Calcium oxalate ureterolith in a cat. Vet. Radiol..

[108] Rishniw M., Weioman J., Hornof W. (1998). Hydrothorax secondary to a perinephric pseudocyst in a cat. Vet. Radiol. Ultrasound.

[109] Robotham G.R. (1999). What is your diagnosis?. J. Am. Vet. Med..

[110] Ticer J.W. (1963). Capsulogenic renal cyst in a cat. J. Am. Vet. Med..

[111] Tidwell A.S., Ullman S.L., Schelling S.H. (1990). Urinorna (para-ureteral pseudocyst) in a dog. Vet. Radiol..

[112] Ochoa V.B., Dibartow S.P., Chew D.J., Westropp J., Carothers M., Biller D. (1999). Perinephric pseudocysts in the cat: A retrospective study and review of the literature. J. Vet. Intern. Med..

[113] Hill T.P., Odesnik B.J. (2000). Omentalisation of perinephric pseudocysts in a cat. J. Small Anim. Pract..

[114] Borisov A.V. (1964). Lymphatic capillaries and blood vessels of milky spots in the human greater omentum. Fed. Proc. Transl. Suppl..

[115] González Domínguez M., Hernandez C., Maldonado-Estrada J. (2010). Protective compromise of great omentum in an asymptomatic uterine rupture in a bitch: A case report. Rev. Colomb. Cienc. Pecuarias.

[116] Platell C., Cooper D., Papadimitriou J.M., Hall J.C. (2000). The omentum. World J. Gastroenterol..

[117] Berry S.J., Strandberg J.D., Coffey D.S., Saunders W.J. (1986). Development of canine benign prostatic hyperplasia with age. Prostate.

[118] Polisca A., Troisi A., Fontaine E., Menchetti L., Fontbonne A. (2016). A retrospective study of canine prostatic diseases from 2002 to 2009 at the Alfort Veterinary College in France. Theriogenology.

[119] Smith J. (2008). Canine prostatic disease: A review of anatomy, pathology, diagnosis, and treatment. Theriogenology.

[120] Freitag T., Jerram R.M., Walker A.M., Warman C.G.A. (2007). Surgical management of common canine prostatic conditions. Compend. Contin. Educ. Vet..

[121] Huggins C., Sommer J.L. (1940). Quantitative studies of prostatic secretion. J. Exp. Med..

[122] Bokemeyer J., Peppler C., Thiel C., Failing K., Kramer M., Gerwing M. (2011). Prostatic cavitary lesions containing urine in dogs. J. Small Anim. Pract..

[123] McGill J., Thieman Mankin K.M., Parambeth J.C., Edwards J., Cook A. (2018). Urine-Filled Large Prostatic Cystic Structure in Two Unrelated Male Miniature Dachshunds. J. Am. Anim. Hosp. Assoc..

[124] Hardie E.M., Barsanti J.A., Rawlings C.A. (1984). Complications of prostatic surgery. J. Am. Anim. Hosp. Assoc..

[125] Mullen H., Matthirsen D., Sacvelli D. (1990). Results of Surgery and Postoperative Complications in 92 Dogs Treated for Prostatic Abscessation by a Multiple Penrose Drain Technique. J. Am. Anim. Hosp. Assoc..

[126] Sisson D.D., Hoffer R.E. (1997). Osteocollagenous prostatic retention cyst: Report of a canine case. J. Am. Anim. Hosp. Assoc..

[127] White R., Herrtage M.E., Dennis R. (1987). The diagnosis and management of paraprostatic and prostatic retention cysts in the dog. J. Small Anim. Pract..

[128] Zolton G.M. (1979). Surgical techniques for the prostate. Vet. Clin. N. Am. Small Anim. Pract..

[129] Hoffer R.E., Dykes N.L., Greiner T.P. (1977). Marsupialization as a treatment for prostatic disease. J. Am. Anim. Hosp. Assoc..

[130] Lee J., Lee K.S., Kim C.L., Byeon J.S., Gu N.Y., Cho I.S., Cha S.H. (2017). Effect of donor age on the proliferation and multipotency of canine adipose-derived mesenchymal stem cells. J. Vet. Sci..

[131] Kimura K., Breitbach M., Schildberg F.A., Hesse M., Fleischmann B.K. (2021). Bone marrow CD73+ mesenchymal stem cells display increased stemness in vitro and promote fracture healing in vivo. Bone Rep..

[132] Ode A., Schoon J., Kurtz A., Gaetjen M., Ode J.E., Geissler S., Duda G.N. (2013). CD73/5′-ecto-nucleotidase acts as a regulatory factor in osteo-/chondrogenic differentiation of mechanically stimulated mesenchymal stromal cells. Eur. Cells Mater..

[133] Chung M.T., Liu C., Hyun J.S., Lo D.D., Montoro D.T., Hasegawa M., Li S., Sorkin M., Rennert R., Keeney M. (2013). CD90 (Thy-1)-positive selection enhances osteogenic capacity of human adipose-derived stromal cells. Tissue Eng. Part A.

[134] Nakamura H., Yukita A., Ninomiya T., Hosoya A., Hiraga T., Ozawa H. (2010). Localization of Thy-1-positive cells in the perichondrium during endochondral ossification. J. Histochem. Cytochem. Off. J. Histochem. Soc..

[135] Vimalraj S., Arumugam B., Miranda P.J., Selvamurugan N. (2015). Runx2: Structure, function, and phosphorylation in osteoblast differentiation. Int. J. Biol. Macromol..

[136] Zhang Q.X., Magovern C.J., Mack C.A., Budenbender K.T., Ko W., Rosengart T.K. (1997). Vascular endothelial growth factor is the major angiogenic factor in omentum: Mechanism of the omentum mediated angiogenesis. J. Surg. Res..

[137] Saifzadeh S., Pourreza B., Hobbenaghi R., Naghadeh B.D., Kazemi S. (2009). Autogenous greater omentum, as a free nonvascularized graft, enhances bone healing: An experimental nonunion model. J. Investig. Surg..

[138] Piermattei D., Johnson K.A., Piermattei D.L., Johnson K.A. (2004). The Hindlimb. An Atlas of Surgical Approaches to the Bones and Joints of the Dog and Cat.

[139] Baltzer W.I., Cooley S., Warnock J.J., Nemanic S., Stieger-Vanagas S.M. (2015). Augmentation of radius and ulna diaphyseal fracture repair in toy breed dogs using free autogenous omental graft. Vet. Comp. Orthop. Traumatol..

[140] Ree J.J., Baltzer W.I., Nemanic S. (2018). Randomized, controlled, prospective clinical trial of autologous greater omentum free graft versus autogenous cancellous bone graft in radial and ulnar fractures in miniature breed dogs. Vet. Surg..

[141] Levy Y., Miko I., Hauck M., Mathesz K., Furka I., Orda R. (1998). Effect of omental angiogenic lipid factor on revascularization of autotransplanted spleen in dogs. Eur. Surg. Res..

[142] Silverman K.J., Lund D.P., Zetter B.R., Lainey L.L., Shahood J.A., Freiman D.G., Folkman J., Barger A.C. (1988). Angiogenic activity of adipose tissue. Biochem. Biophys. Res. Commun..

[143] Konturek S.J., Brzozowski T., Majka I., Pawlik W., Stachura J. (1994). Omentum and basic fibroblast growth factor in healing of chronic gastric ulcerations in rats. Dig. Dis. Sci..

[144] Williams J.K., Carlson G.W., Austin G.E., Austin E.D., Rand R.P., Jurkiewicz M.J. (1996). Short gut syndrome: Treatment by neovascularization of the small intestine. Ann. Plast. Surg..

[145] Song D. (1989). The greater omentum: Its use in the surgical management of severely infected leg soft tissue and bone injuries. Report of two cases. Cent. Afr. J. Med..

[146] Kos J., Nadinic V., Huljev D., Nadinić I., Turić J., Košuta D., Anić T., Babić T., Vnuk D., Kreszinger M. (2006). Healing of bone defect by application of free transplant of greater omentum. Vet. Arh..

[147] Hausman M.R., Schaffler M.B., Majeska R.J. (2001). Prevention of fracture healing in rats by an inhibitor of angiogenesis. Bone.

[148] Rhinelander F.W. (1968). The normal microcirculation of diaphyseal cortex and its response to fracture. J. Bone Jt. Surg. Am..

[149] Singh A.K., Patel J., Litbarg N.O., Gudehithlu K.P., Sethupathi P., Arruda J.A.L., Dunea G. (2008). Stromal cells cultured from omentum express pluripotent markers, produce high amounts of VEGF, and engraft to injured sites. Cell Tissue Res..

[150] Oloumi M.M., Derakhshanfar A., Molaei M.M., Tayyebi M. (2006). The angiogenic potential of autogenous free omental graft in experimental tibial defects in rabbit: Short-term preliminary histopathological study. J. Exp. Anim. Sci..

[151] Street J., Bao M., deGuzman L., Bunting S., Peale F.V., Ferrara N., Steinmetz H., Hoeffel J., Cleland J.L., Daugherty A. (2002). Vascular endothelial growth factor stimulates bone repair by promoting angiogenesis and bone turnover. Proc. Natl. Acad. Sci. USA.

[152] Liang H., Pun S., Wronski T.J. (1999). Bone anabolic effects of basic fibroblast growth factor in ovariectomized rats. Endocrinology.

[153] Matoba Y., Katayama H., Ohami H. (1996). Evaluation of omental implantation for perforated gastric ulcer therapy: Findings in a rat model. J. Gastroenterol..

[154] Rosier R.N., O’Keefe R.J., Hicks D.G. (1998). The potential role of transforming growth factor beta in fracture healing. Clin. Orthop. Relat. Res..

[155] Brockman D.J., Pardo A.D., Conzemius M.G., Cabell L.M., Trout N.J. (1996). Omentum enhanced reconstruction of chronic non-healing wounds in cats: Techniques and clinical use. Vet. Surg..

[156] Gray M.J. (2005). Chronic axillary wound repair in a cat with omentalisation and omocervical skin flap. J. Small Anim. Pract..

[157] Deboer D.J., August J.R. (1991). Nonhealing cutaneous wounds. Consultations in Feline Internal Medicine.

[158] Swain S.F., Henderson R.A. (1990). Small Animal Wound Management.

[159] Dupont C., Menaro Y. (1972). Transposition of the greater omentum for reconstruction of the chest wall. Plast. Reconstr. Surg..

[160] McLean D.H., Bijncke H.J. (1972). Autotransplant of omentum to a large scalp defect with microsurgical revascularisation. Plast. Reconstr. Surg..

[161] Quimby G.F., Diamono D.A., Mor Y., Zaidi Z., Ransley P.G. (1996). Bladder neck reconstruction: Long-term follow-up of reconstruction with omentum and silicone sheath. J. Urol..

[162] Lascelles B.D., White R.A.S. (2001). Combined omental pedicle grafts and thoracodorsal axial pattern flaps for the reconstruction of chronic, nonhealing axillary wounds in cats. Vet. Surg..

